# Modeling the Spatial Distribution of Plateau Pika (*Ochotona curzoniae*) in the Qinghai Lake Basin, China

**DOI:** 10.3390/ani9100843

**Published:** 2019-10-21

**Authors:** Yi-Nan Wu, Yu-Jun Ma, Wen-Ling Liu, Wu-Zhao Zhang

**Affiliations:** 1 Environmental Development Center of the Ministry of Ecology and Environment, Beijing 100029, China; wuyinan@edcmep.org.cn; 2 School of Geography and Planning, Sun Yat-sen University, Guangzhou 510275, China; 3 School of Natural Resources, Faculty of Geographical Science, Beijing Normal University, Beijing 100875, China; liuwl.19b@igsnrr.ac.cn (W.-L.L.); zhangwuzhao@mail.bnu.edu.cn (W.-Z.Z.); 4 Institute of Geographic Sciences and Natural Resources Research, Chinese Academy of Sciences, Beijing 100101, China

**Keywords:** spatial distribution, environmental variable, anthropogenic variable, plateau pika, maxent

## Abstract

**Simple Summary:**

The plateau pika (*Ochotona curzoniae*) is a keystone species on the Qinghai–Tibetan Plateau (QTP). We identified key factors affecting its distribution and predicted it in Qinghai Lake basin by the maximum entropy (Maxent) model at 1-km spatial resolution. Our results showed that the suitable area for plateau pika in Qinghai Lake basin is approximately 3982 km^2^, which occupies 15.8% of the land area in the whole watershed. The distance to road emerged as the most important predictor of distribution patterns of plateau pika, while the soil type was of ancillary importance. With the constraint of human factors, the presence probability of plateau pika in about 1661 km^2^ will increase. These findings indicate that human factors have significant importance for plateau pika’s distribution, and provide evidence to guide plateau pika control in this and other similar regions.

**Abstract:**

The plateau pika (*Ochotona curzoniae*) is a keystone species in the alpine rangeland ecosystem of the Qinghai–Tibetan Plateau. Most previous studies of habitat selection by plateau pika have been conducted at a local microhabitat scale; however, little is known about the relationship between the distribution of plateau pika and macrohabitat factors at broad spatial scales. Using a presence-only ecological niche model (maximum entropy, Maxent), we predicted the distribution of plateau pika in the Qinghai Lake basin based on a set of environmental and anthropogenic variables at 1-km spatial resolution, and identified key macrohabitat factors that contribute to the predictive performance. Our results showed suitable area for plateau pika in the Qinghai Lake basin being approximately 3982 km^2^, which is 15.8% of the land area in the whole watershed. The distance to road emerged as the most important predictor of the distribution patterns of plateau pika, while the soil type was of ancillary importance. Mean air temperature of wettest quarter, distance to resident site and altitude also produced high gains in defining plateau pika’s distribution. A higher predictive accuracy was achieved by the model that combined environmental and anthropogenic variables. With the constraint of human factors, the presence probability of plateau pika in about 1661 km^2^ will increase. These findings demonstrate the impact of human activities on the distribution of plateau pika, and the importance of vegetation reservation for plateau pika control.

## 1. Introduction

Rangeland degradation not only affects pastoralists who rely on healthy grazing lands for their survival, but also influences others who suffer from resultant hydrological disturbances, dust storms and commodity scarcity; therefore, it is a global concern [1]. The Qinghai–Tibetan plateau (QTP) is the world’s highest plateau and one of the most important grazing lands in China. However, the rangeland degradation of the QTP has already lasted for several decades, and the causes are generally considered to be climate change, natural disasters, excessive grazing, soil disturbance by small mammals and so on [2]. Plateau pika (*Ochotona curzoniae*) is one of the main native soil faunas on the QTP, with short limbs, rounded ears and no external tail. It is a small (roughly 170 g) social, burrowing, non-hibernating lagomorph that can attain high population densities [3]. The plateau pika has been traditionally considered as a pest because it competes with livestock for forage and contributes to rangeland degradation [4,5]. However, some ecologists have indicated that the plateau pika helps accelerate the soil nitrogen cycle, increases plant species richness and biodiversity of birds and predatory animals and contributes positively to ecosystem-level dynamics; thus, it has been classified as a keystone species and an ecosystem engineer on the QTP [3,6]. Practically, the population density of plateau pika is an important index for evaluating its ecological function [7,8]. Appropriate population density of plateau pika could promote forage growth and maintain the ecosystem stability as an allogenic engineer [9,10]. Thus, the population and distribution of plateau pika is very important for rangeland management and ecosystem sustainable development on the QTP. 

Field survey was widely implemented in previous research to study the population and distribution of plateau pika at the local microhabitat scale. It has been proven that the main factors affecting plateau pika’s habitat selection include topographic position, soil character, water distance, shrub cover ratio and height of broad leaf vegetation [11]. Zhang et al. also indicated that plateau pika prefer places with lower soil moisture and looser soil, such as the slope and floodplain areas [12]. In general, these studies concentrated on the plateau pika’s habitat selection at a small scale. However, making plenty of sample plots on the whole QTP is not easy obviously, due to the harsh weather, limited roads, time, cost and the steep mountains.

Moreover, it is still a controversy what the role human activities play on the blast and distribution of plateau pika [4,13]. On one hand, human activities such as overgrazing may accelerate the degradation of rangeland, offering a potential suitable habitat for plateau pika [14,15], while Zhou et al. revealed that the road has played some barrier effect on the genetic interaction between the plateau pikas that inhabited the two sides of the Qinghai–Tibet Highway [16]. On the other hand, chemical and biological technology has been used to reduce the population of plateau pika year by year; however, it would recover rapidly [3,17]. Thus, it is important to reveal the influence of multiple factors (including meteorological conditions, soil properties, plant characters, human activities, etc.) on the population and distribution of plateau pika by models [18,19]. 

Species distribution models (SDMs) are numerical tools that predict species distribution by combining observations of species occurrence or abundance with environmental estimates [20]. With the location of a species occurrence and the corresponding values of varied environmental variables extracted from spatial databases, it is possible to fit a model to show the relation between location and environmental variables [20]. After setting up the model, it can be used to predict the distribution of species in the entire region. A variety of SDMs are available to predict potentially suitable habitat for a species, such as maximum entropy (Maxent), generalized linear model (GLM), generalized additive model (GAM) and boosted regression tree (BRT). These models have been employed to predict the distribution of many types of organisms, such as herbs [21], shrubs [22], trees [23], insects [24], fish [25], reptiles [26], birds and mammals [27]. More importantly, SDMs can be used to predict the change of species distribution under different scenarios of climate change and human impact [26,28]. However, previous modeling about plateau pika mainly focused on the population dynamics [19,29], and its spatial distribution has rarely been simulated by SDMs.

Thus, this study aims to: (1) Simulate the spatial distribution of plateau pika in the Qinghai Lake basin based on topographic and bioclimatic variables, satellite-derived vegetation indices and anthropogenic variables using the Maxent model; (2) Identify macrohabitat factors affecting the spatial distribution of plateau pika at the watershed scale; (3) Investigate the relationship between human activities and the distribution of plateau pika. The results of this study will benefit the rangeland management and ecosystem status assessment in the Qinghai Lake basin, and other similar regions.

## 2. Materials and Methods

### 2.1. Study Area

**Figure 1:**
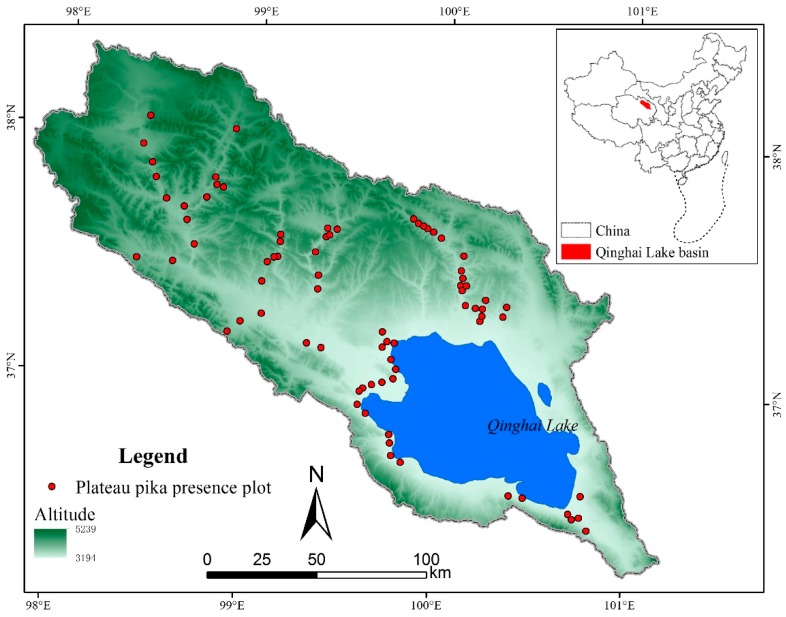
Location of the Qinghai Lake basin and sampling plots of the plateau pika’s distribution.

The Qinghai Lake basin is a closed watershed with an area of 29,661 km^2^, and is located in the northeast of the QTP (Figure 1). Qinghai Lake is the largest inland lake in China with an altitude of 3194 m above sea level (ASL), and it is situated in the semiarid, cold and high-altitude climate zone. The topography of the whole basin extends from northwest to southeast, with the highest altitude reaching more than 5200 m ASL. The mean annual air temperature, precipitation and evaporation between 1960 and 2015 in the Qinghai Lake basin was −0.13 °C, 355 and 925 mm, respectively. The soil types in the Qinghai Lake basin are classified as Typic Cryoboroll and Typic Haploboroll according to USDA taxonomy, or Haplic Kastanozem and Cryi-Haplic Kastanozem according to the World Reference Base for Soil Resources. The Qinghai Lake basin is a typical prairie and includes several different grassland types, such as the warm steppe, alpine grassland and alpine meadow. Since the 1980s, influenced by climate change and human activities, the environmental condition in the basin has degraded intensely, and has led to plenty of ecological problems, such as desertification, loss of fishery resources and bird habitats disappearing [30]. Human population in the whole watershed is about 110 thousand, and this results in few roads and resident sites that are distributed mainly around the Qinghai Lake.

### 2.2. Field Survey

Plateau pika presence data were collected in August from 2015 to 2017 for simulating their distribution and validating the Maxent model. It is difficult to count the plateau pika directly during field survey; therefore, the active burrow density was widely used to indicate their presence and disturbance intensity [7,31]. According to the previous studies, the number of active burrow between 48 ± 8 per ha can be regarded as approximately zero-density, and has little influence on vegetation and soil properties [31,32]. Therefore, plateau pika presence in this study was defined as the active burrow density at more than 40 per ha. During the field survey, it is very difficult to get access to every sample plot generated by software randomly in mountainous area, due to the uneven distribution of roads and pastoralist’s barriers. Thus, sampling plots were mainly designed along the road distributed as uniformly as possible, and their location covered a wide range of vegetation, soil and altitude variation to reflect the environmental condition in the entire watershed. In order to lessen the surveying bias, all the sampling plots were at least 2 km away from the road. Moreover, the distance between each two sample plots was more than 2 km to avoid the spatial autocorrelation [33]. Although the plot of 1 km × 1 km was very hard to measure, the field view at most sample plots was good enough to find plateau pika burrows with visible grassland degradation. Finally, 77 sample plots of plateau pika presence were collected (Figure 1). 

### 2.3. Modeling Approach

Maxent is a general-purposed machine learning method with a precise mathematical formulation used to make predictions for species distribution [34]. It is a presence-only modeling tool so that it only requires presence data rather than presence and absence data. Moreover, Maxent has turned out to be a reliable method when the sample size is relatively small [35]. All environmental variables (see Section 2.4) and anthropogenic variables (see Section 2.5) together with the location of 77 plateau pika presence plots were utilized to create the model. A total of 10,000 background points were randomly created from the entire study area, and 100 replicate runs of Maxent were implemented with the subsample validation method to facilitate the model evaluation and interpretation. Training dataset (n = 38) and test dataset (n = 39) were assigned by Maxent software randomly in each run [36]. The range of 10 km from the road was set as the background point deviation correction area to reduce the sampling error of the sampling plots near the road [37]. Additional parameters were set as the default. 

Then, the jackknife test was applied to diagnose the relative importance of different environmental and anthropogenic variables that may assist in generating the distribution model of plateau pika. The variable with the highest training gain when used in isolation is considered to contain the most predictive ability, whilst the variable that decreases the gain the most when it is omitted appears to have the most information that is not present in the other variables [33,38]. The variables whose contribution was less than 0.5 were excluded when creating the model. 

Both the threshold-dependent method and threshold-independent method, including area under the receiver operating characteristic curve (AUC) and true skill statistic (TSS), were used to evaluate the model performance [36]. The AUC value was obtained from each of the models made by the 100 repetitions with the consensus approach. AUC values range from 0 to 1, with values below 0.5 representing a model that is no better than random and values of 1 representing a model that is highly accurate [39]. Based on the guideline by Swets [40], model performance could be distinguished between worse than random (AUC ≤ 0.5), failed (0.5 < AUC ≤ 0.6), poor (0.6 < AUC ≤ 0.7), fair (0.7 < AUC ≤ 0.8), good (0.8 < AUC ≤ 0.9) and excellent (0.9 < AUC ≤ 1.0). To calculate TSS value, 10,000 pseudo-absence points from the background and 39 presence points from 77 sample plots assigned by Maxent randomly were taken as a test dataset for each run. For each point in this dataset, observed occurrence values (pseudo-absence points = 0, presence points = 1) together with predicted occurrence values derived from Maxent output were recorded. TSS values were calculated for all 11 possible thresholds, and the maximum TSS value indicated an optimized threshold. To compare model performance, only the average of the maximum TSS value for plateau pika prediction is reported. The boundaries of TSS are bad (0 < TSS ≤ 0.2), poor (0.2 < TSS ≤ 0.4), fair (0.4 < TSS ≤ 0.6), good (0.6 < TSS ≤ 0.8) and excellent (0.8 < TSS ≤ 1.0) [34]. The Wilcoxon signed-rank test, a non-parametric equivalent of a paired t-test, was used to compare the model performance in accuracy (i.e., AUC and TSS) [41]. The hypothesis test compares repeated measurements to assess whether their population’s mean rank differs. This process was implemented in R, and a significant difference at *p* < 0.01 between the model scenarios was reported [33]. 

### 2.4. Environmental Variables

#### 2.4.1. Topographic Data

Digital elevation model (DEM) data with 25 m resolution were obtained from the National Geomatics Center of China (http://www.ngcc.cn/). Altitude information was directly extracted from DEM. Slope and aspect information were further calculated in ArcGIS 10.2 (ESRI, Redlands, CA, USA) using a surface tool. Topographic Wetness Index (TWI), a function of both the slope and the upstream contributing area per unit width orthogonal to the flow direction, was used to represent soil moisture in this study due to the lack of high resolution soil moisture products. All topographic layers were resampled to the pixel size of 1 km × 1 km using bilinear algorithm.

#### 2.4.2. Soil Data

Soil type data in the Qinghai Lake basin were obtained from the China Soil Type map (1:1,000,000) [42]. It includes the information of soil type and soil subtype represented by soil code.

Soil properties data, including bulk density, the content of clay, the content of sand, organic carbon and coarse fragments volumetric, were derived from the SoilGrids system (http://www.soilgrids.org/). It is a global soil data product with a resolution of 1 km generated by ISRIC—World Soil Information, and is produced using state-of-the-art model-based geostatistical methods [43]. It contains soil information from 0 to 2 m. In this study, information from 0 to 15 cm was adopted considering the depth of plateau pika burrows [44].

#### 2.4.3. Vegetation Data

Vegetation distribution data were obtained from the Vegetation Atlas of China (1:1,000,000) [45]. It reflects not only the vegetation type and condition, but also the horizontal zonality and the vertical zonal distribution. Both categorical maps were converted from polygon to raster with 1 km resolution using the nearest neighbor algorithm to keep consistent with the data above.

In this study, both the Normalized Difference Vegetation Index (NDVI) and the Enhanced Vegetation Index (EVI) time series in 2015 were downloaded from the MODIS/Terra vegetation indices 16-day products (MOD13A2) at 1 km resolution (http://modis.gsfc.nasa.gov/). Two tiles (h25v05, h26v05) of MODIS data were collected to cover the whole study area. In order to decrease the possible noise and keep the data accurate, the satellite images were smoothed using the Savitzky–Golay filter in the TIMESAT program before calculating the indices [46]. At last, the statistics of the NDVI and EVI for the whole year, including mean value, maximum value, minimum value and standard deviation, were calculated. 

The Leaf Area Index (LAI) data in 2015 were retrieved from the MODIS Leaf Area Index/FPAR 8 day products (MCD15A2) at 1 km resolution (https://lpdaac.usgs.gov/). Further, the LAI information was extracted from the LAI band, and was processed using same procedures as NDVI and EVI.

#### 2.4.4. River Distribution

The distance to water was proved to be important indicator that affects the distribution of plateau pika [11]. Therefore, river distribution datasets (1:250,000) in the Qinghai Lake basin were obtained from the Cold and Arid Regions Science Data Center Database (http://westdc.westgis.ac.cn/). They were further used to create distance map by Euclidean Distance tools in ArcGIS 10.2, clipped by the study area and resampled to 1 km resolution to be the same as the other data. 

#### 2.4.5. Bioclimatic Data

Bioclimatic data were downloaded from the WorldClim database (http://www.worldclim.org/). This dataset represents biologically meaningful variables for characterizing species distribution at a large spatial scale [23]. In this study, 19 climatic layers with a spatial resolution of 1 km were used (Table 1) [33]. 

#### 2.4.6. Land Surface Temperature

Land surface temperature (LST) is a key indicator of the earth surface heat condition and has obvious effects on vegetation growth. In this study, the LST time series in 2015 was derived from the MODIS Land Surface Temperature and Emissivity 8 day products (MOD11A2) at 1 km resolution (http://modis.gsfc.nasa.gov/). The information for LST at daytime and nighttime was extracted from the LST-day and LST-night band, respectively. Then the images were projected and smoothed using same procedures as NDVI and EVI.

### 2.5. Anthropogenic Variables

The population density in the Qinghai Lake basin was less than 5 person/km^2^, and most of the people concentrated in the lake rim zone, especially along the road. Therefore, both the road and the resident site distribution datasets (1:250,000) were obtained from the Cold and Arid Regions Science Data Center Database (http://westdc.westgis.ac.cn/). Then, they were used to create a distance map, and were resampled to 1 km resolution with the same method as river distribution.

### 2.6. Selection of Variables

At last, all above data were converted to ASCII file acceptable in Maxent. In summary, 58 layers were made as input data (Supplementary Material Table S1). We ran the Maxent model with all the variables first to indicate the extent of relevance to the plateau pika’s distribution. In order to reduce the effect of highly correlated variables, the Pearson correlation among different layers was checked using ENMtools and a correlation coefficient (Supplementary Material Table S2) [47]. If two variables were high correlated (r > 0.75 or r < −0.75), the one that had lower grades in the jackknife test was deleted. Finally, 12 variables remained to assemble the Maxent model (Table 2). 

## 3. Results

### 3.1. Plateau Pika’s Distribution in Qinghai Lake Basin

The receiver operating characteristic curve was far away from the diagonal, which indicates random prediction. Both the AUC (0.90) and the TSS (0.67) value represented a good performance of the plateau pika’s distribution modeling by Maxent. Therefore, the modeling results can be further used to analyze the spatial distribution of plateau pika in the Qinghai Lake basin. 

**Figure 2:**
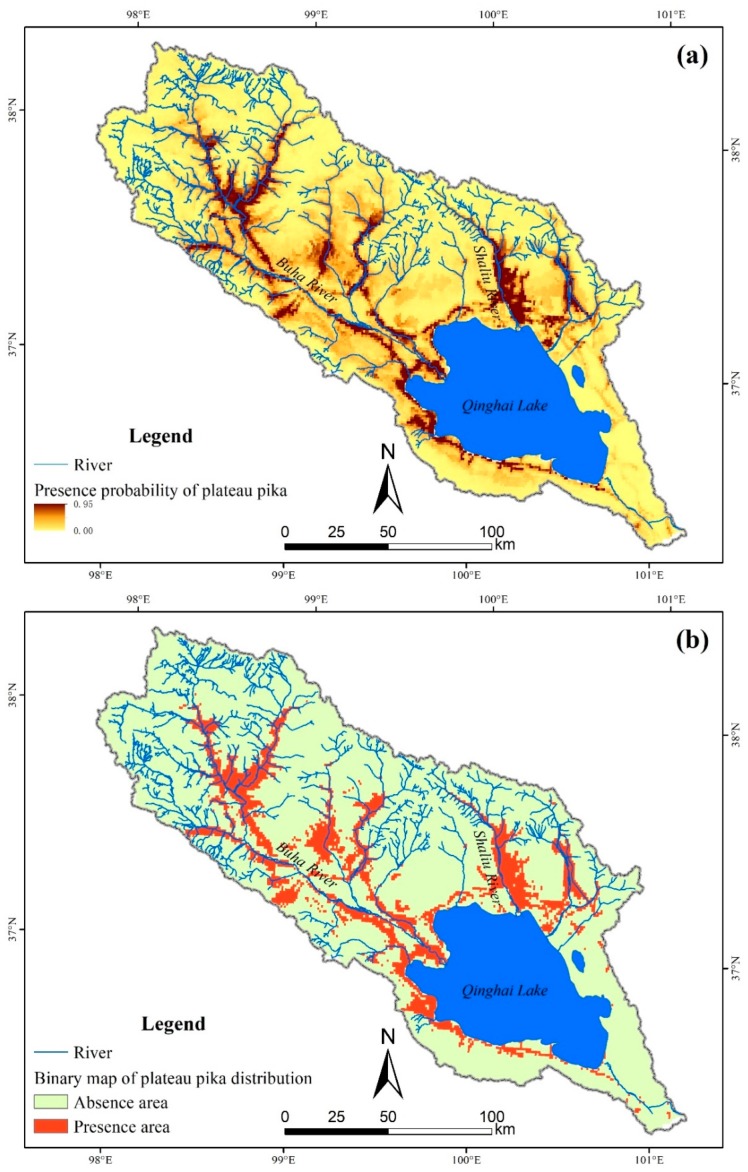
Presence probability map (**a**) and binary map (**b**) of plateau pika’s distribution in the Qinghai Lake basin.

Plateau pika mainly distributes along the river and road in the Qinghai Lake basin, especially in the west of Qinghai Lake, the upstream of the Buha River and the middle stream of the Shaliu River (Figure 2a). According to the calculated TSS results, maximum test sensitivity plus specificity with maximum TSS value was chosen as the optimized threshold to convert the logistical probability map to binary map [48], and the threshold probability to reclassify plateau pika presence and absence was 0.1734. The binary map shows that the suitable area for plateau pika is approximately 3982 km^2^ in the Qinghai Lake basin, which occupies 15.8% of land area in the whole watershed (Figure 2b). 

### 3.2. Macrohabitat Factors Affecting Plateau Pika’s Distribution

**Figure 3:**
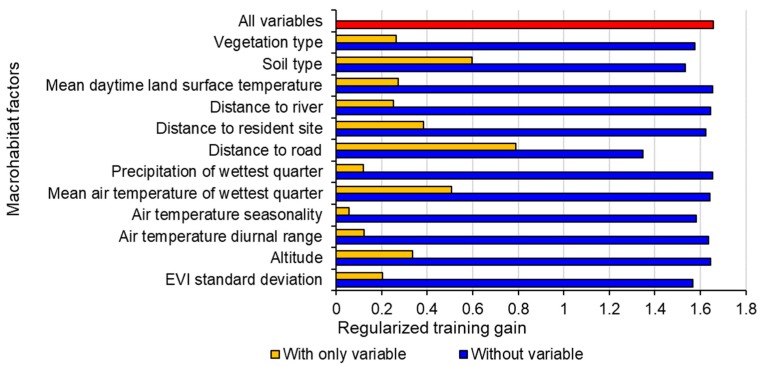
Importance of macrohabitat factors in modeling the distribution of plateau pika in the Qinghai Lake basin. “With only variable” indicates the results of the model when a single variable is run; “Without variable” indicates the effect of removing a single variable from the full model; “All variables” indicates the results of the model when all variables are run.

The results of the jackknife test in Maxent indicate that distance to road contributes most to the model, and the soil type results in the secondary gain for the model (Figure 3). Mean air temperature of the wettest quarter, the distance to resident site and the altitude also produce high gains (>0.3) for defining plateau pika’s distribution. Other environmental variables, including mean daytime land surface temperature, vegetation type, distance to river, EVI standard deviation, air temperature diurnal range, precipitation of wettest quarter and air temperature seasonality, have relatively little gain for the model. 

**Figure 4:**
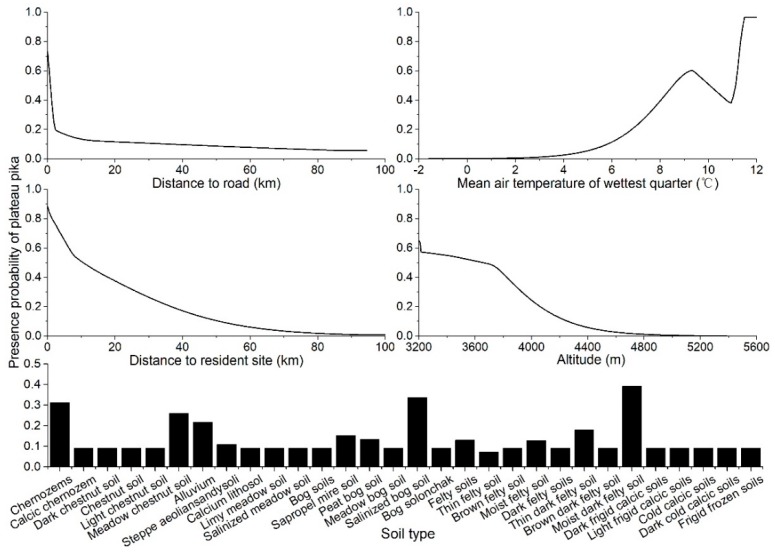
Response curves illustrating the relationship between the presence probability of plateau pika and macrohabitat factors in the Qinghai Lake basin.

According to the response curves, for the most important five macrohabitat factors, plateau pikas probably occur (probability ≥ 0.5) in areas where the distance to the road is lower than 0.9 km, the mean air temperature of the wettest quarter is between 8.5 °C and 10.0 °C or higher than 11.1 °C, the distance to resident site is lower than 10.5 km and the altitude is lower than 3655 m (Figure 4). Moist dark felty soil, salinized bog soil, chernozems, meadow chestnut soil and alluvium are the suitable soil types for plateau pika living. The highest presence probability responded to these factors when a certain value was reached. For example, plateau pikas are most likely to occur in areas near roads and resident sites, where the altitude is around 3200 mm and where the mean air temperature of the wettest quarter is around 12 °C. 

### 3.3. Impact of Human Activities on Plateau Pika’s Distribution

**Figure 5:**
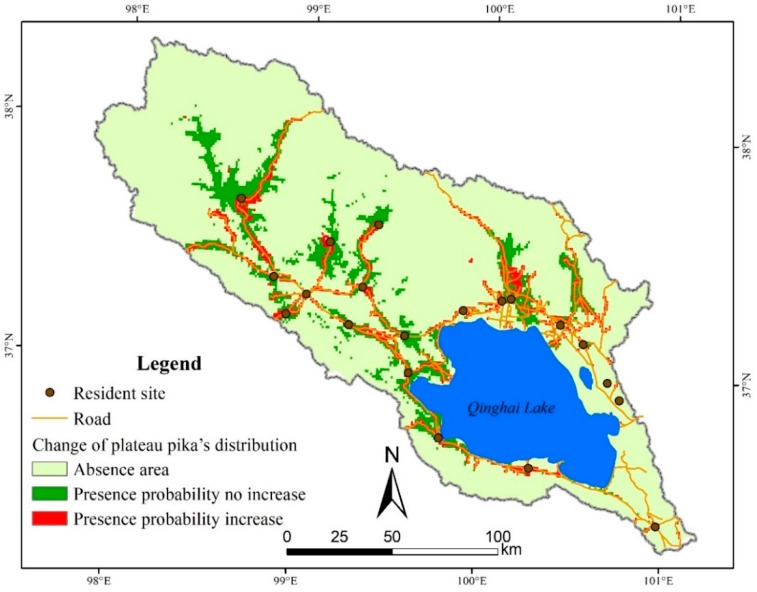
Change of plateau pika’s distribution in the Qinghai Lake basin with and without human factors.

In order to investigate the relation between human activities and the distribution of plateau pika in Qinghai Lake basin, we made another Maxent model without human factors (distance to road, and distance to resident site). All parameters were set as same as the previous model, and the performance of this new model is fair with 0.86 of mean AUC and 0.62 of mean TSS. The presence probability of plateau pika in about 1661 km^2^ will increase with human factors, i.e., the distribution range of plateau pika may extend 72% with the influence of human activities, compared with the modeling results without human factors (Figure 5). Therefore, more roads and resident sites may expand the active scope of plateau pika. 

Furthermore, a Wilcoxon paired test was used to test whether the difference between these two models (with and without human factors) is significant [49]. All the mean values of training AUC, test AUC and TSS have significant difference between the modeling results with and without human factors (*p* < 0.01), indicating the importance of human factors to predict plateau pika’s distribution in the Qinghai Lake basin (Table 3). All indices showed a decreasing trend when removing human factors from of input data. Without the constraint of human factors, the performance of the Maxent modeling of plateau pika’s distribution in the Qinghai Lake basin dropped from good to fair. 

## 4. Discussion

### 4.1. The Importance of Human Activities to Plateau Pika’s Distribution

Our result clearly show the significant importance of human factors on plateau pika’s habitat selection. Even though “distance to road” and “distance to resident site” only represents part of the human activities intensity, they were proven to be suitable factors for predicting the plateau pika’s distribution in the Qinghai Lake basin. Both of these two variables showed a negative relation with the plateau pika’s distribution, because, firstly, the construction of the roads and resident sites needs the destruction of the vegetation and the excavation of the soil, which will reduce vegetation coverage and soil hardness, and create a more suitable habit for plateau pika [5,12]. This result indicates that the expanding of plateau pika may be partly the results of human disturbance, and it is extremely important to protect and reserve the vegetation during the construction of roads and resident sites for plateau pika control. 

Besides distance to road and resident site, grazing also has important effect on the plateau pika’s distribution. Previous studies have shown that the average carrying capacity of plateau pika is higher on degraded meadow than that on undegraded meadow, because vegetation shortens with the increase of grazing intensity [29]. Moreover, high levels of livestock grazing could benefit plateau pika populations by increasing erosion and creating additional burrowing opportunities for plateau pikas [14]. Li et al. also revealed that livestock grazing could increase the risk of plateau pika outbreaks, due to it reducing the vegetation height and diminishing the plateau pika predation risk [15]. Lots of previous studies demonstrated that proper plateau pika density is beneficial to the entire ecosystem [9,10], but the fragment of suitable habitat for plateau pika may cause its population to rise and accelerate rangeland degradation, because dispersal is an infrequent event for plateau pika [3]. 

### 4.2. Environment Factors Affecting Plateau Pika’s Distribution

The response curve of soil type showed that the soil with a porous structure, high humidity and rich organic materials is fit for plateau pika survival, which was consistent with Niu et al. [50]. Obviously, soft and relatively moist soil is convenient for plateau pika to make burrows, and organic materials will ensure the vegetation condition meets its demands of food. Plateau pika maybe prefers the habitats with warm conditions because high temperatures may be helpful for its growth, breeding and thermogenic adjustment in this high-altitude region [51]. Moreover, the temperature in the rainy season also controls the vegetation living condition in this semiarid alpine basin, and further affects the plateau pika’s distribution indirectly. 

Higher standard deviation of EVI indicates higher rangeability of grassland, which means that degraded grassland is more suitable for the plateau pika’s distribution, and this is consistent with Fang and Shi [52]. Shi and Yu also showed that the ideal plateau pika’s habitat is the grassland with loose soil, low cover and low height of plant communities [31]. Moreover, the intertwined root system may make the upper layer of the meadow habitat difficult to penetrate by plateau pika [3]. Distance to water, reflecting the difficulty for plateau pika to get water, shows a negative relation with the probability of plateau pika presence, which is consistent with Wang et al. [11]. 

### 4.3. Modeling Plateau Pika’s Distribution with Maxent

The sample size will affect the accuracy of Maxent results sensitively, and a large sample size always indicates high AUC values [53]. In this study, the AUC value could reach 0.92, while the TSS value was 0.55 before removing presence data with spatial autocorrelation. After removing these presence data with spatial autocorrelation, mean AUC value decreased to 0.90 but TSS value increased to 0.67. Thus, AUC value could not evaluate the performance of species distribution model alone, which has been discussed a lot before [54]. How to select the proper environmental factors is another challenge when using species distribution models. Highly correlated environmental variables have been proven to affect the interpretation of the predictor’s response [55], but the effect on the overall accuracy of the model is rarely reported. Our results revealed that using highly correlated environmental variables will affect the response curve of the model intensively, but the overall accuracy of the model will not be influenced a lot. 

Previous studies revealed that plateau pika would increase rapidly in numbers after the winter; even if its abundance was reduced greatly in early spring due to widespread poisoning, the population could recover over the following summer [17]. Therefore, the combination of its spatial distribution and population density need further investigation. On the other hand, due to the limitation of uneven roads and pastoralist’s barriers, our field sampling plots were all along the road (>2 km), and whether the predicted result in the uninvestigated areas is reasonable needs to be validated by more field testing. With the development of aerial photographing, it will become a useful tool to study the spatial distribution of plateau pika on a broader scale [56].

## 5. Conclusions

Plateau pika’s distribution in the Qinghai Lake basin was simulated successfully with a Maxent model based on the combination of its presence data, environmental data and anthropogenic data. Approximately 15.8% of land is suitable area for plateau pika in the Qinghai Lake basin, and it mainly distributed in the west of Qinghai Lake, the upstream of the Buha River and the middle stream of the Shaliu River. The distance to road emerged as the most important predictor of distribution patterns of plateau pika, while the soil type was of ancillary importance. With the construction of roads and resident sites, the presence probability of plateau pika in about 1661 km^2^ will increase. These findings indicate that human factors have significant importance for plateau pika’s distribution, and they provide evidence to guide plateau pika control in this and other similar regions.

## Figures and Tables

**Table 1:** Bioclimatic data downloaded from the WorldClim database.

Variable	Abbreviation	Units
Annual mean air temperature	bio1	°C
Air temperature diurnal range (mean of monthly (maximum–minimum))	bio2	°C
Isothermality (bio2/bio7)	bio3	Dimensionless
Air temperature seasonality (standard deviation)	bio4	°C
Maximum air temperature of warmest month	bio5	°C
Minimum air temperature of coldest month	bio6	°C
Air temperature annual range (bio5–bio6)	bio7	°C
Mean air temperature of wettest quarter	bio8	°C
Mean air temperature of driest quarter	bio9	°C
Mean air temperature of warmest quarter	bio10	°C
Mean air temperature of coldest quarter	bio11	°C
Annual precipitation	bio12	mm
Precipitation of wettest month	bio13	mm
Precipitation of driest month	bio14	mm
Precipitation seasonality (coefficient of variation)	bio15	Dimensionless
Precipitation of wettest quarter	bio16	mm
Precipitation of driest quarter	bio17	mm
Precipitation of warmest quarter	bio18	mm
Precipitation of coldest quarter	bio19	mm

**Table 2:** Remaining variables selected by Pearson correlation and jackknife test for assembling the Maxent model.

Remained Variable	Abbreviation
EVI standard deviation	2015evi_std
Altitude	altitude
Air temperature diurnal range (mean of monthly (maximum–minimum))	bio2
Air temperature seasonality (standard deviation)	bio4
Mean air temperature of wettest quarter	bio8
Precipitation of wettest quarter	bio16
Distance to resident site	dis_to_resident
Distance to river	dis_to_river
Distance to road	dis_to_road
Mean daytime land surface temperature	lst_day_mean
Soil type	soil_type
Vegetation type	vegetation_type

**Table 3:** Wilcoxon paired test values showed significant difference of plateau pika’s distribution in the Qinghai Lake basin with and without human factors.

Model	Training AUC		Test AUC		TSS	
	Mean	SD	Mean	SD	Mean	SD
With human factors	0.9539	0.0069	0.8954	0.0337	0.6723	0.0800
Without human factors	0.9355	0.0071	0.8608	0.0300	0.6222	0.0728

AUC: area under the receiver operating characteristic curve; TSS: true skill statistic; SD: standard deviation.

## Supplementary Materials

The following are available online at https://www.mdpi.com/2076-2615/9/10/843/s1, Supplementary Table S1: All variables for the first running of Maxent model to indicate the extent of relevance to plateau pika’s distribution; Supplementary Table S2: The Pearson correlation coefficient among different variables for the modelling of plateau pika’s distribution.
